# The mevalonate pathway is an actionable vulnerability of *t*(4;14)-positive multiple myeloma

**DOI:** 10.1038/s41375-020-0962-2

**Published:** 2020-07-14

**Authors:** Joseph Longo, Petr Smirnov, Zhihua Li, Emily Branchard, Jenna E. van Leeuwen, Jonathan D. Licht, Benjamin Haibe-Kains, David W. Andrews, Jonathan J. Keats, Trevor J. Pugh, Suzanne Trudel, Linda Z. Penn

**Affiliations:** 1 Princess Margaret Cancer Centre, University Health Network, Toronto, ON Canada; 2 Department of Medical Biophysics, University of Toronto, Toronto, ON Canada; 3 Vector Institute, Toronto, ON Canada; 4 Division of Hematology/Oncology, University of Florida Health Cancer Center, Gainesville, FL USA; 5 Ontario Institute for Cancer Research, Toronto, ON Canada; 6 Department of Computer Science, University of Toronto, Toronto, ON Canada; 7 Sunnybrook Research Institute, Toronto, ON Canada; 8 Translational Genomics Research Institute, Phoenix, AZ USA

**Keywords:** Cancer metabolism, Targeted therapies

## Abstract

Multiple myeloma (MM) is a plasma cell malignancy that is often driven by chromosomal translocations. In particular, patients with *t*(4;14)-positive disease have worse prognosis compared to other MM subtypes. Herein, we demonstrated that *t*(4;14)-positive cells are highly dependent on the mevalonate (MVA) pathway for survival. Moreover, we showed that this metabolic vulnerability is immediately actionable, as inhibiting the MVA pathway with a statin preferentially induced apoptosis in *t*(4;14)-positive cells. In response to statin treatment, *t*(4;14)-positive cells activated the integrated stress response (ISR), which was augmented by co-treatment with bortezomib, a proteasome inhibitor. We identified that *t*(4;14)-positive cells depend on the MVA pathway for the synthesis of geranylgeranyl pyrophosphate (GGPP), as exogenous GGPP fully rescued statin-induced ISR activation and apoptosis. Inhibiting protein geranylgeranylation similarly induced the ISR in *t*(4;14)-positive cells, suggesting that this subtype of MM depends on GGPP, at least in part, for protein geranylgeranylation. Notably, fluvastatin treatment synergized with bortezomib to induce apoptosis in *t*(4;14)-positive cells and potentiated the anti-tumor activity of bortezomib in vivo. Our data implicate the *t*(4;14) translocation as a biomarker of statin sensitivity and warrant further clinical evaluation of a statin in combination with bortezomib for the treatment of *t*(4;14)-positive disease.

## Introduction

Translocations between the immunoglobulin heavy chain (*IGH*) gene on chromosome 14 and putative oncogenes on other chromosomes are hallmarks of multiple myeloma (MM) [1, 2]. In particular, translocations between *IGH* and chromosome 4 affect ~15% of MM patients, and are associated with poor progression-free and overall survival [3–5]. Despite recent improvements in the management of *t*(4;14)-positive disease, it remains difficult to treat and is largely incurable [6]. This is partly due to the fact that the precise driver of *t*(4;14)-positive MM remains debated.

**Fig. 1:**
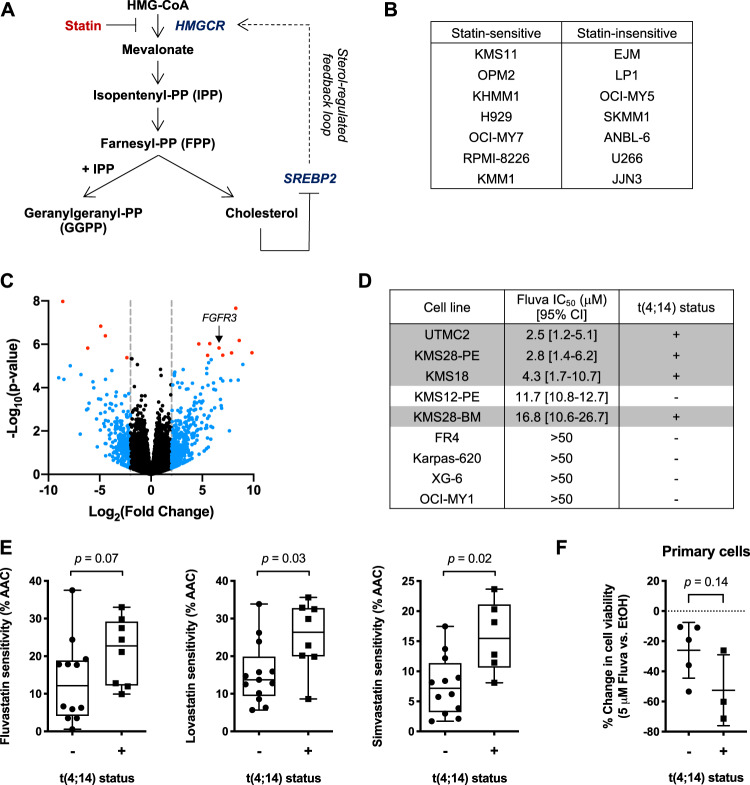
The *t*(4;14) translocation is associated with statin sensitivity in MM. **a** Schematic representation of the MVA pathway and its sterol-regulated feedback loop. Statins inhibit the rate-limiting enzyme of the MVA pathway, HMGCR. Statin-mediated cholesterol depletion activates the SREBP2 transcription factor, which induces genes involved in MVA metabolism, including *HMGCR*. **b** List of lovastatin-sensitive and insensitive MM cell lines previously characterized by Wong et al. [10]. **c** Differential gene expression analysis between statin-sensitive and insensitive MM cell lines listed in (**b**). The blue and red dots represent differentially expressed genes with a log_2_(fold change) >2 or <−2. The genes in red further pass the adjusted *p* value cut-off when corrected for multiple testing (Bonferroni correction). Black dots represent genes with no significant difference in expression between statin-sensitive and insensitive MM cell lines. **d** Sensitivity to fluvastatin determined by MTT assays following 48 h of treatment. Fluvastatin IC_50_ value and 95% confidence interval (CI) for each cell line are shown. The *t*(4;14) translocation status is indicated and *t*(4;14)-positive cell lines are shaded in gray. **e** Sensitivity to fluvastatin, lovastatin, and simvastatin in *t*(4;14)-positive or negative MM cell lines mined from the CTRPv2 database. Percent area above the drug dose-response curve (% AAC) values are plotted as a box plot with whiskers representing minimum and maximum values. A higher % AAC indicates greater drug sensitivity (unpaired, two-tailed Wilcoxon rank-sum test comparing *t*(4;14)-positive and negative MM cell lines). **f** Primary plasma cells from *t*(4;14)-positive or negative patients were cultured in the presence of 5 μM fluvastatin or ethanol as a solvent control. After 72 h, cells were labeled with PE-conjugated anti-CD138 and FITC-conjugated Annexin V, and then cells were analyzed by flow cytometry. The percent change in primary CD138+ cell viability is plotted. The data are represented as the mean ± SD, *p* value = 0.14 (unpaired, two-tailed Wilcoxon rank-sum test).

Evidence supports that statins, common cholesterol-lowering drugs, have anti-cancer activity. Statins are inhibitors of HMG-CoA reductase (HMGCR), the rate-limiting enzyme of the mevalonate (MVA) pathway, which is responsible for the synthesis of cholesterol and non-sterol isoprenoids that are crucial for cell growth and survival (Fig. 1a) [7]. We, and others, have demonstrated that statins induce apoptosis in a subset of MM cell lines by directly inhibiting HMGCR, indicating that some MM cells are dependent on the MVA pathway for survival [8–11]. These preclinical data are supported by recent retrospective analyses, where statin use was associated with reduced MM-specific mortality [12, 13]. Moreover, phase I/II trials in patients with relapsed/refractory MM have reported promising responses when a statin was combined with standard-of-care therapies in some, but not all, patients [14, 15]. Collectively, these data suggest that these safe and inexpensive drugs may be effective anti-MM agents, but highlight the need for a predictive biomarker of statin sensitivity for patient stratification.

Using transcriptomic and drug sensitivity data, we show here that MM cells driven by a *t*(4;14) translocation are particularly sensitive to statin-induced apoptosis. We further demonstrate that fluvastatin can synergize with bortezomib to induce apoptosis in *t*(4;14)-positive MM cells. Taken together, our data implicate the *t*(4;14) translocation as a biomarker of statin sensitivity and warrant further evaluation of the statin-bortezomib combination for the treatment of *t*(4;14)-positive disease.

## Materials and methods

### Cell lines, primary cells, and reagents

Cell lines were cultured as previously described [10]. All other cell lines were cultured in Iscove’s Modified Dulbecco’s Medium (Gibco) with 10% fetal bovine serum, 100 units/mL penicillin, and 100 mg/mL streptomycin. Cell lines were routinely confirmed to be mycoplasma-free (MycoAlert, Lonza). Primary human MM cells were obtained from bone marrow aspirates or peripheral blood draws from consenting MM patients with approval from the University Health Network’s Research Ethics Board. Isolated cells were processed and analyzed as previously described [11]. See “Supplemental Information” for reagent details.

### Cell viability and Annexin V assays

MTT assays were performed as previously described [11]. Cell lines were seeded at 10,000–20,000 cells/well in 96-well plates and treated with 0–50 μM fluvastatin for 48 h. For Annexin V assays, 750,000 cells were seeded in six-well plates and treated as indicated. Cells were processed using the FITC Annexin V Apoptosis Detection Kit (BD Biosciences) as per the manufacturer’s protocol.

### Animal experiments

In all, 7–9-week-old female NOD/SCID mice were injected with 5 million cells subcutaneously in the flank in a 1:1 mixture with Matrigel (Corning). Mice were treated as indicated with fluvastatin (orally, in phosphate-buffered saline) and/or bortezomib (intraperitoneally, in saline). Animal experiments were approved by the University Health Network’s Research Ethics Board and performed in accordance with Canadian Council on Animal Care regulations.

### Supplemental methods

Method descriptions for quantitative RT-PCR (qRT-PCR), immunoblotting, live-cell imaging, RNA interference, RNA sequencing (RNA-seq), and differential expression analysis are available in “Supplemental Information.”

## Results

### The *t*(4;14) chromosomal translocation is associated with statin sensitivity in MM

We previously screened a panel of 17 MM cell lines for their response to lovastatin treatment [10]. We observed a dichotomized response, where approximately half of the cell lines underwent apoptosis in response to lovastatin exposure and the other half remained viable [10]. Using these drug sensitivity data in combination with basal RNA-seq data, we performed differential gene expression analysis comparing seven statin-sensitive and seven insensitive cell lines (Fig. 1b). One of the differentially expressed genes between the two groups was fibroblast growth factor receptor 3 (*FGFR3*), where *FGFR3* was more highly expressed in statin-sensitive cell lines (Fig. 1c and Table S1). FGFR3 expression is deregulated in ~15% of MM patients as the result of a translocation between chromosome 4 and the *IGH* locus at chromosome 14q32, which places *FGFR3* under the control of the 3′ *IGH* enhancer [16, 17]. Given our observation that statin-sensitive MM cells express high levels of *FGFR3*, we decided to evaluate whether *t*(4;14) translocation status was associated with statin sensitivity.

We built an independent validation panel of MM cell lines and evaluated their sensitivity to fluvastatin. We chose to evaluate fluvastatin instead of lovastatin, as lovastatin has been reported to have HMGCR-independent activities through its interaction with P-glycoprotein [18, 19] and the proteasome [20]. In contrast, fluvastatin is more specific to HMGCR and is less likely to participate in unwanted drug–drug interactions compared to many of the other statins [18, 21]. We treated cells with a range of fluvastatin concentrations and compared the IC_50_ values derived from the dose-response curves after 48 h of treatment. Consistent with our hypothesis, cells that were *t*(4;14)-positive had physiologically achievable [22, 23], low micromolar IC_50_ values compared to *t*(4;14)-negative cell lines, which were predominantly insensitive to fluvastatin at the concentrations evaluated (Fig. 1d).

We next mined publicly available drug sensitivity data from the Cancer Therapeutics Response Portal version 2 (CTRPv2) database using PharmacoDB [24, 25]. In CTRPv2, data for three statins (fluvastatin, lovastatin, and simvastatin) were available. We extracted statin sensitivity data as area above the drug dose-response curve (AAC) for all MM cell lines that were evaluated (20, 21, and 18 for fluvastatin, lovastatin, and simvastatin, respectively) (Fig. 1e). When stratified based on *t*(4;14) translocation status, MM cell lines that were *t*(4;14)-positive had higher AAC values for all three statins compared to *t*(4;14)-negative cell lines, indicating greater drug sensitivity (Fig. 1e). Ten MM cell lines in the CTRPv2 database were unique from those that we independently validated for statin sensitivity [10] (Fig. 1d), and the remaining cell lines were concordant in their response to statin treatment between data sets.

To establish whether fluvastatin could also kill primary cells derived from patients with *t*(4;14)-positive MM, we exposed primary cells to fluvastatin ex vivo. After 72 h of treatment, viable MM cells (CD138+/Annexin V−) were quantified by flow cytometry. Consistent with our cell line data, a trend toward increased sensitivity was observed in primary cells derived from *t*(4;14)-positive patients (Figs. 1f and S1).

Finally, we grew *t*(4;14)-positive NCI-H929 (hereafter referred to as H929) cells as xenografts in mice. Once tumors reached ~200 mm^3^, the mice were randomized to receive treatment with vehicle control, 20 or 50 mg/kg/day fluvastatin. Consistent with our in vitro data, we observed a dose-dependent decrease in tumor volumes over time in response to treatment (Fig. S2). Taken together, these data clearly demonstrate an association between *t*(4;14) status and statin sensitivity in MM.

### Fluvastatin sensitivity in *t*(4;14)-positive MM cells is independent of FGFR3 and MMSET

Since we observed higher *FGFR3* expression in statin-sensitive MM cell lines and an association between *t*(4;14) translocation status and statin sensitivity, we next evaluated whether the deregulated expression of *FGFR3* in *t*(4;14)-positive cells was necessary for the greater statin sensitivity observed in these cells. We generated KMS11 sublines (statin sensitive and *t*(4;14)-positive) that express doxycycline-inducible short hairpin RNAs (shRNAs) against *FGFR3* or a non-targeting shRNA control. Treatment of these sublines with doxycycline for 48 h was sufficient to reduce FGFR3 expression, but did not alter fluvastatin sensitivity (Fig. S3). In addition to FGFR3, the histone methyltransferase MMSET (*NSD2*) is also deregulated as a result of the *t*(4;14) translocation; however, depletion of MMSET in KMS11 cells, with previously characterized shRNAs [26] (Fig. S4), similarly had no effect on fluvastatin-induced apoptosis (Fig. S3).

A small proportion of *t*(4;14)-positive MM cells also have FGFR3-activating mutations [17]. Only three cell lines in our panel (KMS11, OPM2, and KMS18) had a mutation in FGFR3, and no correlation was observed between FGFR3 mutation status and statin sensitivity (data not shown). While mutations in MMSET are not as common in MM, aberrant activation of MMSET occurs as a result of a point mutation in acute lymphoblastic leukemia (ALL) cells [27]; however, we observed no differences in statin sensitivity between wild-type and E1099K MMSET-expressing ALL cell lines (Fig. S5). Collectively, these data support that statin sensitivity is independent of FGFR3 and MMSET.

### The *t*(4;14) translocation is a novel and independent biomarker of statin sensitivity

We previously reported that sensitivity to lovastatin in MM was associated with an impaired sterol-regulated feedback response in statin-sensitive cells [11]. When cells are exposed to a statin, the depletion of intracellular cholesterol triggers the activation of the sterol regulatory element-binding protein (SREBP) family of transcription factors (Fig. 1a). The SREBPs subsequently induce the expression of genes involved in MVA and sterol metabolism, including *HMGCR* and *HMGCS1*, to restore homeostasis. In a subset of cell lines, this feedback loop fails to engage in response to statin treatment, and therefore the working model is that these cells are unable to compensate for the loss of crucial MVA-derived metabolites, and thereby undergo apoptosis.

To test whether *t*(4;14)-positive cells were sensitive to statins because of impaired feedback regulation of the MVA pathway, we evaluated the induction of *HMGCR* and *HMGCS1* in a panel of *t*(4;14)-positive and negative MM cell lines following fluvastatin treatment. No association between *t*(4;14) translocation status and impaired feedback regulation of the MVA pathway was observed (Fig. S6), providing evidence that the *t*(4;14) translocation and impaired feedback regulation of the MVA pathway are independent predictors of statin sensitivity in MM.

### Fluvastatin induces the integrated stress response (ISR) in *t*(4;14)-positive cells

To better understand this unique dependency of *t*(4;14)-positive MM cells on the MVA pathway, we performed RNA-seq to identify genes that were perturbed in response to fluvastatin treatment in *t*(4;14)-positive MM cell lines. Gene ontology analysis of fluvastatin-perturbed genes revealed a number of upregulated and downregulated biological processes (Fig. S7). Fluvastatin-downregulated genes were largely involved in processes such as RNA splicing and ribosome biogenesis, whereas a number of fluvastatin-upregulated genes were involved in response to stress. In particular, genes involved in the ISR, including *ATF4*, *ATF3*, and *DDIT3* (also known as *CHOP*), were induced following fluvastatin treatment. The ISR is activated in response to various stressors, including endoplasmic reticulum (ER) stress and nutrient deprivation [28]. These stresses result in the activation of kinases that converge on the phosphorylation of eIF2α at serine 51 [28]. Phosphorylation of eIF2α leads to the global attenuation of mRNA translation, while selectively allowing for the translation of a subset of proteins to aid in cell recovery and survival, including the transcription factor ATF4 [28]. ATF4 then induces the expression of a number of downstream target genes, including *ATF3*, *PPP1R15A* (also known as *GADD34*), and *CHOP*, the latter of which induces apoptosis if the initiating stress remains unresolved [28].

Currently, one of the most important classes of anti-MM therapeutics is the proteasome inhibitors, including bortezomib [29], which remain important agents for the improved management of *t*(4;14)-positive disease [30]. Bortezomib is known to activate the eIF2α-ATF4 signaling axis via the induction of ER stress [31], and studies have shown that activation of this signaling axis is important for the proapoptotic activity of bortezomib [32–34]. Hence, we decided to further validate whether inhibition of the MVA pathway by statins could result in activation of the ISR in *t*(4;14)-positive cells.

**Fig. 2:**
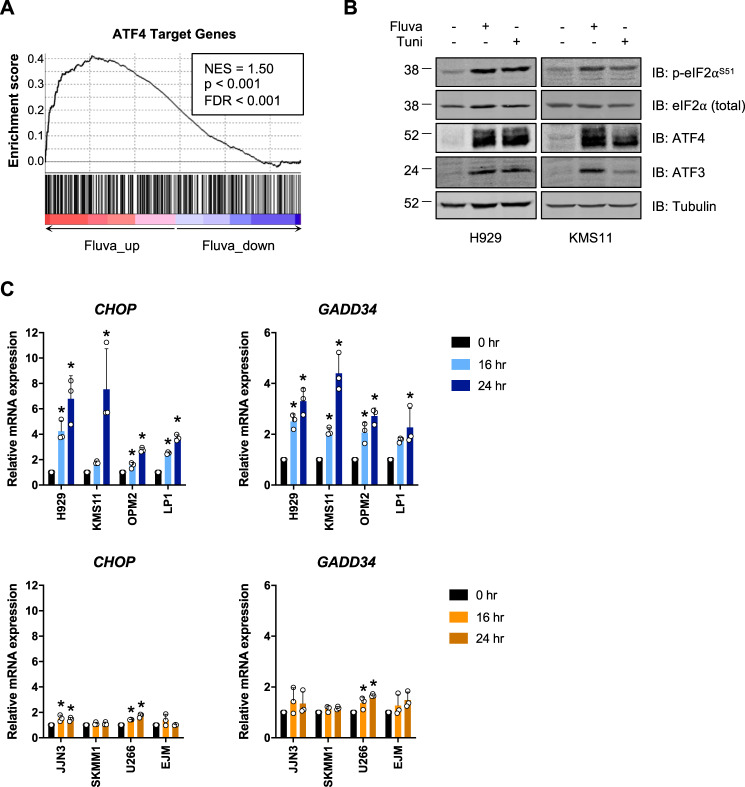
Fluvastatin induces the integrated stress response (ISR) in *t*(4;14)-positive MM cells. H929 and KMS11 cells were treated with 2 μM fluvastatin or ethanol as a solvent control for 24 h, after which RNA was isolated and RNA-seq was performed. **a** Gene set enrichment analysis of ATF4 target genes in a gene list of fluvastatin-perturbed genes, ranked by fold change between fluvastatin-treated and ethanol-treated cells. **b** H929 and KMS11 cells were treated with solvent controls, 4 μM fluvastatin or 0.5 μg/mL tunicamycin for 24 h, after which protein was isolated and immunoblotting was performed to evaluate phosphorylated eIF2α, ATF4, and ATF3 expression. **c** *t*(4;14)-positive cells (H929, KMS11, OPM2, and LP1; in blue) and *t*(4;14)-negative cells (JJN3, SKMM1, U266, and EJM; in orange) were treated with ethanol control (0 h fluvastatin) or 4 μM fluvastatin for 16 or 24 h, after which RNA was isolated for qRT-PCR. The ATF4 target genes *CHOP* and *GADD34* were evaluated and expression was normalized to *RPL13A*. The data are represented as the mean + SD, *n* = 3, *p < 0.05 (one-way ANOVA with Bonferroni’s multiple comparisons test, where each group was compared to the ethanol control).

We performed gene set enrichment analysis to compare our list of fluvastatin-perturbed genes to a published list of ATF4 target genes [35]. Of the 472 previously reported ATF4 targets, 307 were differentially expressed between solvent control and fluvastatin-treated *t*(4;14)-positive cells, with a significant enrichment of ATF4 target genes among the genes upregulated in response to fluvastatin treatment (Fig. 2a). Moreover, fluvastatin treatment resulted in the phosphorylation of eIF2α and induction of both ATF4 and ATF3 expression by immunoblotting, which was comparable to the increase observed following treatment with tunicamycin, a known inducer of ER stress (Fig. 2b). We then evaluated mRNA expression of two ATF4 target genes, *CHOP* and *GADD34*. We observed a time-dependent increase in both *CHOP* and *GADD34* expression in *t*(4;14)-positive cell lines following fluvastatin exposure; however, in *t*(4;14)-negative cells, these genes were either not induced or, in some lines, weakly induced compared to *t*(4;14)-positive lines (Fig. 2c).

To evaluate whether fluvastatin activated the ISR via induction of ER stress in *t*(4;14)-positive cells, we evaluated the expression of *GRP78* and *XBP1* splicing, which are induced together with eIF2α-ATF4 signaling as part of the unfolded protein response [36]. The concentration of fluvastatin that induced ATF4 target gene expression in *t*(4;14)-positive cells had no effect on *GRP78* expression or *XBP1* splicing compared to tunicamycin, suggesting that fluvastatin induces the ISR via a mechanism independent of ER stress (Fig. S8).

### Geranylgeranyl pyrophosphate (GGPP) rescues statin-induced apoptosis and ISR activation in *t*(4;14)-positive cells

**Fig. 3:**
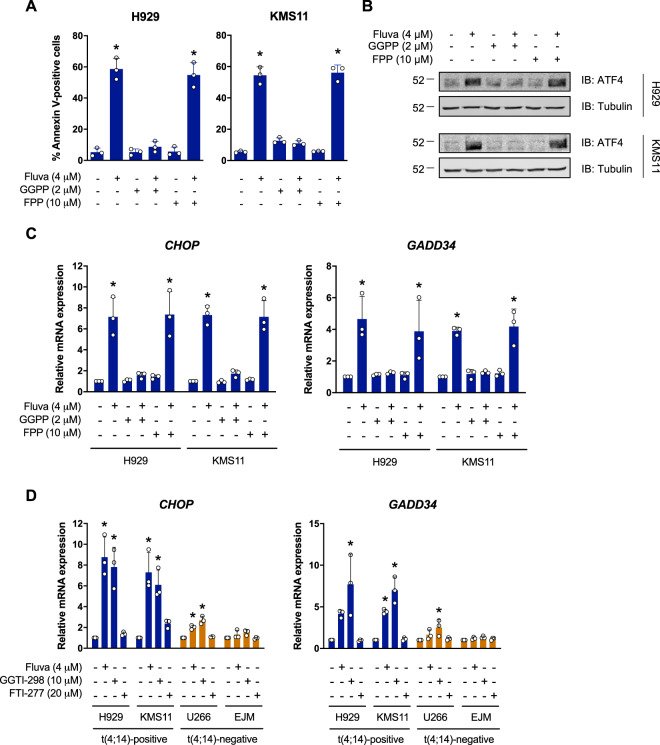
Fluvastatin-induced apoptosis and ISR activation can be rescued by exogenous GGPP. **a** *t*(4;14)-positive H929 or KMS11 cells were treated with 4 μM fluvastatin ± 2 μM GGPP or 10 μM FPP for 48 h, and apoptosis was determined by Annexin V staining. The data are represented as the mean + SD, *n* = 3, *p < 0.05 (one-way ANOVA with Bonferroni’s multiple comparisons test, where each group was compared to the solvent controls group). **b** H929 or KMS11 cells were treated with 4 μM fluvastatin ± 2 μM GGPP or 10 μM FPP for 24 h, after which **b** protein was isolated and immunoblotting was performed to evaluate ATF4 expression or **c** RNA was isolated for qRT-PCR. The ATF4 target genes *CHOP* and *GADD34* were evaluated and expression was normalized to *RPL13A*. The data are represented as the mean + SD, *n* = 3, *p < 0.05 (one-way ANOVA with Bonferroni’s multiple comparisons test, where each group was compared to the solvent controls group). **d** H929, KMS11, U266, and EJM cells were treated with solvent controls, 4 μM fluvastatin, 10 μM GGTI-298, or 20 μM FTI-277 for 24 h, after which RNA was isolated for qRT-PCR. The ATF4 target genes *CHOP* and *GADD34* were evaluated and expression was normalized to *RPL13A*. The data are represented as the mean + SD, *n* = 3, *p < 0.05 (one-way ANOVA with Bonferroni’s multiple comparisons test, where each group was compared to the solvent controls group).

Statin-induced apoptosis is known to be an on-target response, as exogenous MVA can fully rescue cell viability [10, 22, 37, 38]. Studies that have attempted to rescue statin-induced apoptosis with other MVA-derived metabolites have found that exogenous GGPP, and sometimes farnesyl pyrophosphate (FPP), can rescue statin-induced cell death [10, 38, 39]. Consistent with these data, the addition of GGPP, but not FPP, was sufficient to fully rescue fluvastatin-induced apoptosis in *t*(4;14)-positive H929 and KMS11 cells (Fig. 3a), indicating that these cells depend on the MVA pathway for the synthesis of GGPP.

Since fluvastatin treatment induced a robust ISR in *t*(4;14)-positive cells, we next evaluated whether this response was similarly on-target and circumvented by exogenous GGPP. Indeed, exogenous GGPP, but not FPP, fully rescued fluvastatin-induced ATF4 expression (Fig. 3b) and upregulation of *CHOP* and *GADD34* (Fig. 3c), suggesting that GGPP depletion triggers the ISR in *t*(4;14)-positive cells.

FPP and GGPP are used as substrates for the posttranslational prenylation (farnesylation and geranylgeranylation, respectively) of various proteins, which enables their proper membrane localization and function [40]. GGPP can further serve as a precursor for the synthesis of ubiquinone and dolichols [7]. Our lab previously demonstrated that statin-induced MM cell death can be phenocopied by a geranylgeranylation inhibitor (GGTI), which specifically blocks the transfer of GGPP onto target proteins [10]. Hence, one working model is that statin-sensitive MM cells depend on GGPP for protein prenylation. To test whether inhibition of protein geranylgeranylation could similarly phenocopy statin-induced activation of the ISR in *t*(4;14)-positive cells, we treated cells with a GGTI or farnesyltransferase inhibitor (FTI), which blocks the transfer of FPP onto target proteins, as a negative control. Treatment of *t*(4;14)-positive H929 and KMS11 cells with GGTI-298, but not FTI-277, was sufficient to induce *CHOP* and *GADD34* expression (Fig. 3d). In contrast, neither GGTI-298 nor FTI-277 were able to induce the ISR in *t*(4;14)-negative EJM cells, and GGTI-298 only weakly induced *CHOP* and *GADD34* expression in *t*(4;14)-negative U266 cells (Fig. 3d). Collectively, these data support that *t*(4;14)-positive cells are dependent on the MVA pathway for the production of GGPP, and suggest that GGPP may be required, at least in part, for protein prenylation.

### Fluvastatin and bortezomib induce a robust ISR in *t*(4;14)-positive cells and synergize to induce apoptosis

**Fig. 4:**
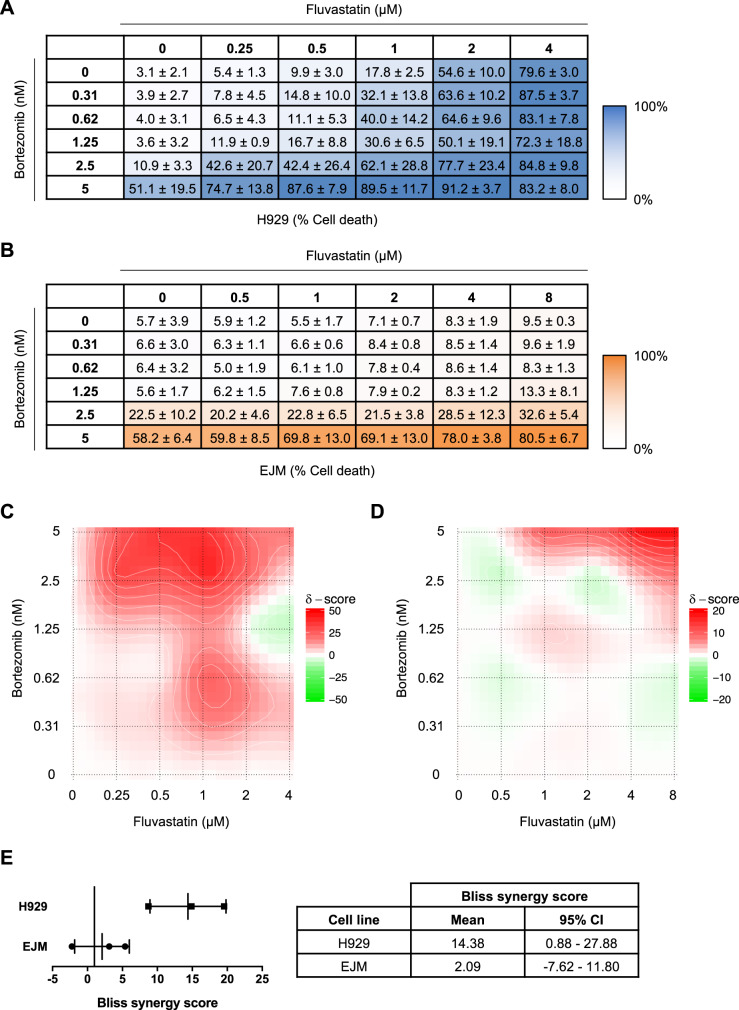
Fluvastatin and bortezomib synergize to induce *t*(4;14)-positive MM cell death. **a** H929 and **b** EJM cells were treated with fluvastatin (ranging from 0 to 4 μM for H929 and 0 to 8 μM for EJM) or bortezomib (ranging from 0 to 5 nM for both cell lines) as single agents or in combination for 48 h. The dose-response matrices, as determined by live-cell imaging and quantification of % dead cells, are representative of three independent experiments and depict the mean % dead cells ± SD. The mean % dead cell values for each dose combination were used to compute Bliss synergy scores for each cell line. Synergy plots for **c** H929 and **d** EJM cells are shown, where red represents synergy and green represents antagonism. **e** The Bliss synergy scores from the three independent experiments are plotted for H929 and EJM cells. The data are represented as the mean ± SD. The mean Bliss synergy score and 95% confidence interval (CI) of the mean for each cell line are reported in the table.

Given that bortezomib also induces the ISR, we sought to determine whether combining these two clinically approved agents would potentiate apoptosis. We treated *t*(4;14)-positive H929 and *t*(4;14)-negative EJM cells with a range of fluvastatin and/or bortezomib concentrations for 48 h, after which cells were stained with Hoechst-33342 (DNA dye), TMRE (marker of active mitochondria), and Annexin V (marker of apoptotic cells). The stained cells were then imaged by confocal microscopy (Fig. S9), and the images were subjected to linear classification analysis to determine the percentage of dead cells for each treatment condition (Fig. 4a, b). The dose-response matrices were then used to compute synergy scores using SynergyFinder [41]. We observed synergy between physiologically achievable [22, 23] concentrations of fluvastatin and low nanomolar concentrations of bortezomib in H929 cells, where fluvastatin reduced the concentration of bortezomib required to induce MM cell death (Fig. 4c, e). This same effect was not observed in *t*(4;14)-negative EJM cells, where synergy with bortezomib was only observed at higher concentrations of fluvastatin (Fig. 4d, e).

**Fig. 5:**
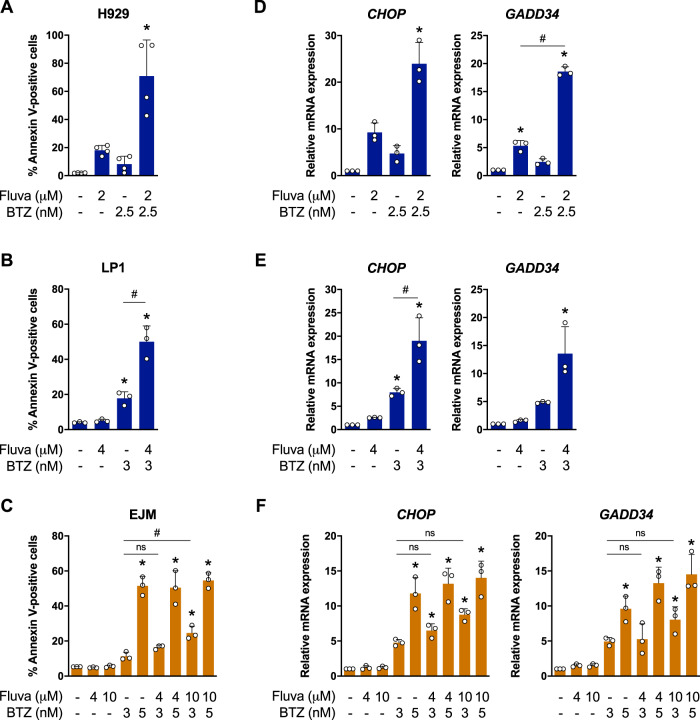
Fluvastatin and bortezomib cooperate to induce the ISR and cell death in *t*(4;14)-positive MM cells. **a** H929, **b** LP1, or **c** EJM cells were treated with solvent controls, fluvastatin or bortezomib (BTZ) at the indicated concentrations for 48 h, and apoptosis was determined by Annexin V staining. The data are represented as the mean + SD, *n* = 3–4, *p < 0.05 (one-way ANOVA with Tukey’s multiple comparisons test, or Kruskal–Wallis test with Dunn’s multiple comparisons test (**a**), where each group was compared to the solvent controls group), ^#^*p* < 0.05 (one-way ANOVA with Tukey’s multiple comparisons test, comparing the two indicated groups). **d** H929, **e** LP1, or **f** EJM cells treated with solvent controls, fluvastatin, or BTZ at the indicated concentrations for 24 h, and RNA was isolated for qRT-PCR. The ATF4 target genes *CHOP* and *GADD34* were evaluated and expression was normalized to *RPL13A*. The data are represented as the mean + SD, *n* = 3, *p < 0.05 (one-way ANOVA with Tukey’s multiple comparisons test, where each group was compared to the solvent controls group), ^#^*p* < 0.05 (one-way ANOVA with Tukey’s multiple comparisons test, comparing the two indicated groups).

To further validate these results, we assayed MM cell lines treated with fluvastatin and/or bortezomib for Annexin V staining by flow cytometry and ISR activation by qRT-PCR (Fig. 5). Consistent with our synergy data, the combination of fluvastatin and bortezomib, at doses that had a minimal effect when used as single agents, resulted in significantly enhanced apoptosis in both H929 and LP1 cells (Fig. 5a, b). This was associated with a greater increase in *CHOP* and *GADD34* expression when the two drugs were used in combination, compared to their effects on the ISR as single agents (Fig. 5d, e). Intriguingly, H929 cells have an impaired sterol-regulated feedback response and are highly sensitive to statins, whereas LP1 cells have a very robust feedback response that reduces their sensitivity to statins [11, 42] (Fig. S6). This reveals that the statin-bortezomib combination can induce apoptosis in *t*(4;14)-positive cells independent of feedback regulation of the MVA pathway. Moreover, bortezomib had no effect on fluvastatin-induced expression of *HMGCR* or *HMGCS1* in LP1 cells (Fig. S10), indicating that bortezomib cooperates with fluvastatin to induce apoptosis via a mechanism that is independent of SREBP and the sterol-regulated feedback response of the MVA pathway.

In contrast, we did not observe the same level of potentiation when EJM cells were treated with fluvastatin and bortezomib; however, a slight, but statistically significant, increase in apoptosis was observed when a higher dose (10 μM) of fluvastatin was combined with 3 nM bortezomib (Fig. 5c). Consistent with the apoptosis data, no additional increases in *CHOP* or *GADD34* expression were observed when EJM cells were treated with bortezomib in combination with fluvastatin (Fig. 5f). Notably, bortezomib alone was sufficient to induce *CHOP* and *GADD34* expression in EJM cells, highlighting that bortezomib and fluvastatin converge on the ISR via distinct mechanisms (Fig. 5f). Collectively, these data demonstrate that the addition of fluvastatin to bortezomib augments activation of the ISR in *t*(4;14)-positive MM cells, and that physiologically achievable concentrations of fluvastatin can synergize with bortezomib to induce *t*(4;14)-positive MM cell death.

### Fluvastatin potentiates bortezomib activity in a *t*(4;14)-positive tumor model

**Fig. 6:**
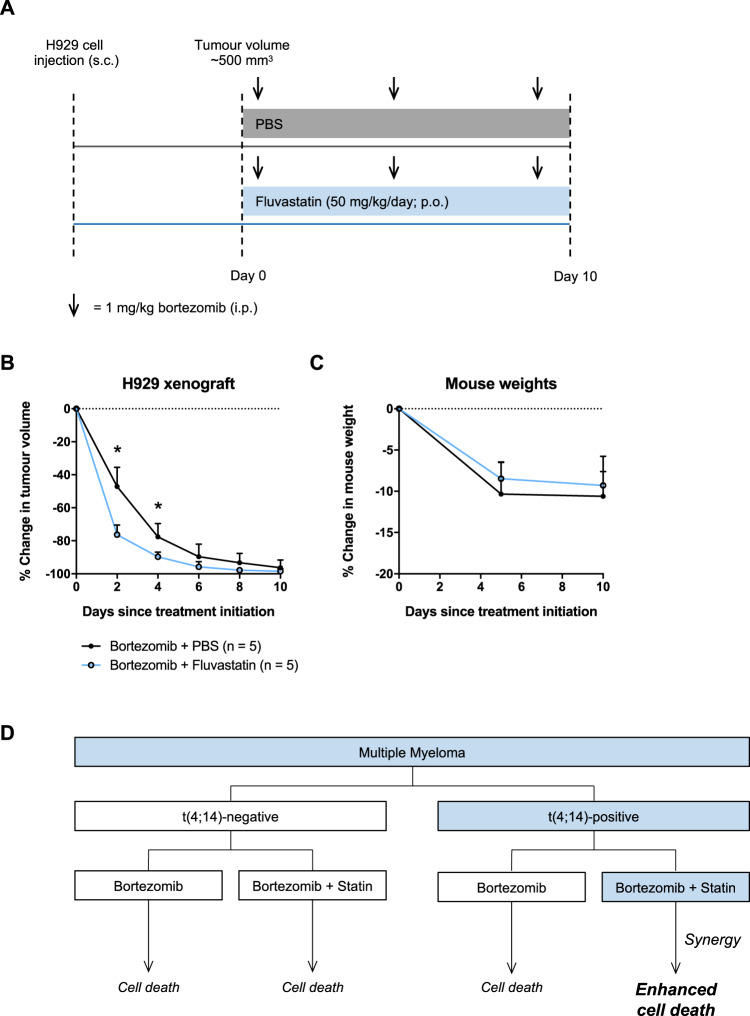
Fluvastatin potentiates bortezomib activity in a *t*(4;14)-positive tumor model. **a** NOD/SCID mice were injected with 5 million H929 cells subcutaneously (s.c.) in the flank. Once tumor volumes reached ~500 mm^3^, the mice were randomized to receive either bortezomib in combination with phosphate-buffered saline (PBS; vehicle control) or 50 mg/kg fluvastatin. Bortezomib was delivered twice per week at 1 mg/kg via intraperitoneal (i.p.) injection, up to a total of three doses. Fluvastatin was resuspended in PBS and delivered daily via oral gavage (per os; p.o.). Tumor measurement assessments were not blinded. **b** Percent change in tumor growth over time. The data are represented as the mean + SD, *n* = 5 mice per treatment group. *p < 0.05 (unpaired, two-tailed Wilcoxon rank-sum test). **c** Percent change in mouse body weight over the course of treatment. **d** Schematic diagram detailing the potential for statins to be used in combination with bortezomib for the personalized treatment of *t*(4;14)-positive MM.

Given that fluvastatin and bortezomib synergized to induce cell death in *t*(4;14)-positive cells in vitro, we decided to evaluate this drug combination in vivo. We grew *t*(4;14)-positive H929 cells as xenografts in mice. Once tumor volumes reached ~500 mm^3^, the mice were randomized to receive treatment with a clinically relevant dose of bortezomib (1 mg/kg) as a single agent or in combination with 50 mg/kg fluvastatin (Fig. 6a). A 50 mg/kg dose of fluvastatin in a mouse is approximately equivalent to 4 mg/kg in a human [43], which has been shown to be well-tolerated [44]. Consistent with our in vitro data, fluvastatin treatment significantly sensitized H929 tumors to bortezomib, with no overt added toxicity (Fig. 6b, c).

## Discussion

Statins have been shown to induce apoptosis in subsets of cancer cells across a wide variety of tumor types [10, 19, 22, 38, 45]; however, biomarkers that predict which cancer cells will respond to statin treatment are lacking. Our lab previously characterized a panel of 17 MM cell lines for their sensitivity to lovastatin, but at the time was unable to correlate statin sensitivity with any single MM-associated genetic abnormality [10]. In that study, five cell lines were *t*(4;14)-positive, four of which were characterized as lovastatin sensitive. In our current study, we took an unbiased, genome-wide approach to interrogate differentially expressed transcripts between statin-sensitive and insensitive MM cell lines. Through this approach, we independently identified that high *FGFR3* expression was associated with increased statin sensitivity in MM, which prompted us to evaluate the *t*(4;14) translocation as a possible biomarker. Indeed, we observed a clear association between the *t*(4;14) translocation and statin sensitivity in MM, which we validated by analyzing an independent panel of cell lines and publicly available drug sensitivity data. In total, 36 MM cell lines were evaluated, 11 of which were *t*(4;14)-positive.

Although the precise driver of *t*(4;14)-positive MM remains unclear, we evaluated whether oncogenes known to be deregulated in *t*(4;14)-positive disease functionally contribute to statin sensitivity. Interestingly, we found that statin sensitivity was independent of FGFR3 and MMSET (Fig. S3). While other genes flank the genomic breakpoints at 4p16, their expression is less consistently deregulated in *t*(4;14)-positive cells [46]. It is possible that a complex interaction between multiple deregulated genes, on chromosome 4 or elsewhere, confers statin sensitivity in *t*(4;14)-positive MM. Alternatively, this translocation could be a surrogate marker for another co-occurring genetic abnormality. For example, the *t*(4;14) translocation has been associated with deletions of chromosome 13q and mutations in *PRKD2* and *DIS3* [47, 48]. Further research is needed to identify the driver(s) of statin sensitivity in *t*(4;14)-positive MM cells.

We previously showed that statin sensitivity is associated with an impaired ability to induce the expression of MVA pathway genes following statin treatment [11, 22]. This was not the case here, as we demonstrated that statin sensitivity in *t*(4;14)-positive cells was not simply due to a lack of feedback regulation of the MVA pathway. While some *t*(4;14)-positive lines failed to induce the expression of *HMGCR* and *HMGCS1* in response to fluvastatin exposure, others significantly upregulated the expression of these genes (Fig. S6). We previously demonstrated that inhibiting this feedback response with the drug dipyridamole sensitizes MM cells, including *t*(4;14)-positive cells, to statin-induced apoptosis [42]. In the present study, we identified that fluvastatin and bortezomib also synergize to induce apoptosis in *t*(4;14)-positive cells (Figs. 4 and 5). In contrast to dipyridamole, the statin-bortezomib interaction was independent of feedback regulation of the MVA pathway, as apoptosis was potentiated in both feedback-impaired (e.g., H929) and feedback-intact (e.g., LP1) *t*(4;14)-positive cell lines, and bortezomib did not function to inhibit the sterol-regulated feedback loop of the MVA pathway (Fig. S10).

We showed that *t*(4;14)-positive MM cells are dependent on the MVA pathway for the synthesis of GGPP, and that the depletion of GGPP triggers the ISR in these cells. Moreover, co-treatment with bortezomib, a drug already used to treat patients with *t*(4;14)-positive MM, augments this response and synergizes with statin treatment to induce *t*(4;14)-positive cell death. While statin-mediated activation of the ISR has been reported in other cancer cell types [49, 50], our study uncovered a clinically relevant biomarker capable of identifying MM cells that will induce this proapoptotic mechanism in response to statin treatment.

Although GGPP is important for various biological processes [7], we demonstrated that treatment of *t*(4;14)-positive MM cells with a GGTI phenocopies statin treatment and induces the ISR, thus implicating protein prenylation in *t*(4;14)-positive cell survival. Hundreds of proteins are predicted to be prenylated in mammalian cells [40, 51], and therefore it is not surprising that attempts to rescue statin-induced apoptosis with select individual prenylated proteins have largely failed [10]. More recent evidence supports a “class effect,” where statins deplete GGPP and hinder the prenylation of multiple proteins important for cell survival [52]. Further investigation is required to elucidate the importance of protein prenylation in *t*(4;14)-positive MM, and delineate the relationship between GGPP metabolism and the ISR.

Our findings have important clinical implications. Our in silico and in vitro data strongly support an association between the *t*(4;14) translocation and statin sensitivity in MM; however, further clinical validation is necessary prior to advancing statins to clinical trials in *t*(4;14)-positive patients. Unfortunately, no transgenic or patient-derived models of *t*(4;14)-driven MM have been developed. As an alternative approach, we evaluated the response of primary patient-derived MM cells to fluvastatin treatment ex vivo. While we demonstrated a promising trend toward increased statin sensitivity in *t*(4;14)-positive samples (Figs. 1f and S1), this association was not statistically significant in our small patient cohort. Regrettably, at the time of revision, the on-going COVID-19 pandemic limited our access to additional primary samples. A more rigorous analysis of statin sensitivity in primary MM cells should be the focus of future studies. Given that statins are already widely prescribed for cholesterol management, a retrospective comparison of *t*(4;14)-positive patient outcomes between statin users and nonusers may also validate our preclinical results.

To date, few studies have evaluated statins in combination with bortezomib in MM [14, 53]. Notably, our study is the first to describe a molecular subtype of MM in which these two drugs interact synergistically, at physiologically relevant concentrations. These data reveal that *t*(4;14)-positive patients in particular may benefit from the addition of a statin to their standard treatment regimen, which includes bortezomib (Fig. 6d). Hence, clinical validation of this immediately available drug combination for the treatment of *t*(4;14)-positive MM is warranted.

## Supplementary information

Supplemental Information

## Data Availability

The basal RNA-seq data are a Keats lab resource (https://www.keatslab.org/data-repository). The fluvastatin treatment RNA-seq data were deposited in the Gene Expression Omnibus (GEO) database (GSE152327). The code used to analyze these data can be found at: https://github.com/bhklab/StatinsMM.
