# Supplementary material for: The mevalonate pathway is an actionable vulnerability of t(4;14)-positive multiple myeloma
Source: Leukemia. 2020 Jul 14;35(3):796–808. doi: 10.1038/s41375-020-0962-2 (PMC7359767; doi:10.1038/s41375-020-0962-2)
Supplement: Supplementary file 1 — Supplemental Information [file 41375_2020_962_MOESM1_ESM.pdf]

SUPPLEMENTAL MATERIALS & METHODS

Reagents

Compound	Solvent	Source
Fluvastatin, sodium salt	Ethanol (*in vitro* use)<br>PBS (*in vivo* use)	US Biological (F5277-76)
Bortezomib	DMSO (*in vitro* use)	Sigma (5043140001)
Bortezomib (Velcade)	Saline (*in vivo* use)	UHN Pharmacy
GGTI-298	DMSO	Tocris Biosciences (2430/1)
FTI-277	Water	Tocris Biosciences (2407/1)
Doxycycline hyclate	Water	Sigma (D9891)
FPP ammonium salt	Water	Sigma (F6892)
GGPP ammonium salt solution	*n/a*	Sigma (G6025)

Quantitative RT-PCR (qRT-PCR)

Total RNA was isolated from subconfluent cells using TRIzol Reagent (Invitrogen). cDNA was synthesized from 500 ng RNA using SuperScript III (Invitrogen). qRT-PCR was performed using the ABI Prism 7900HT sequence detection system and TaqMan probes (Applied Biosystems) for: *HMGCR* (Hs00168352), *HMGCS1* (Hs00266810) and *RPL13A* (Hs01578913). To evaluate the expression of ATF4 target genes, the following primer sequences were used at a final concentration of 100 nM per PCR reaction:

Target	Primer sequences (5' to 3')
CHOP	*Forward*: GGAGCATCAGTCCCCCACTT<br>*Reverse*: TGTGGGATTGAGGGTCACATC
GADD34	*Forward*: CCCAGAAACCCCTACTCATGATC<br>*Reverse*: GCCCAGACAGCCAGGAAAT
GRP78	*Forward*: TGACATTGAAGACTTCAAAGCT<br>*Reverse*: CTGCTGTATCCTCTTCACCAGT
XBP1 (spliced)	*Forward*: CGCTTGGGGATGGATGCCCTG<br>*Reverse*: CCTGCACCTGCTGCGGACT
XBP1 (unspliced)	*Forward*: AGTCCGCAGCACTCAGACTACG<br>*Reverse*: TGGCAGGCTCTGGGGAAGGG
RPL13A	*Forward*: CTTCTCGGCCTGTTTCCGTAG<br>*Reverse*: CGAGGTTGGCTGGAAGTACC

Immunoblotting

Membranes were blocked for 1 hour in 5% milk in Tris-buffered saline (TBS)/0.1% Tween-20 (TBS-T) at room temperature, and then probed with the following primary antibodies in 5% milk/TBS-T overnight at 4 °C: FGFR3 (1:200; Santa Cruz, sc-13121), MMSET (1:200; Abcam, ab75359), α-Tubulin (1:3,000; Calbiochem, CP06), Actin (1:3,000; Sigma, A2066), H3K36me2 (1:1,000; Abcam, ab176921), total H3 (1:5,000; Abcam, ab1791), Ku80 (1:3,000; Cell Signaling Technology, #2180), phosphorylated ERK1/2 (T202/Y204) (1:1,000; Cell Signaling Technology, #4370), total ERK1/2 (1:1,000; Cell Signaling Technology, #4695), phosphorylated eIF2α (S51) (1:1,000; Cell Signaling Technology, #3398), total eIF2α (1:1,000; Cell Signaling Technology, #9722), ATF4 (1:500; Abcam, ab23760), ATF3 (1:200; Santa Cruz, sc-81189). Primary antibodies were detected using IRDye-conjugated secondary antibodies and the Odyssey Classic Imaging System (LI-COR Biosciences).

Live-cell imaging and analysis

H929 and EJM cells were seeded at approximately 3,000-4,000 cells/well in a CellCarrier-384 Ultra Microplate (PerkinElmer). Cells were treated with the indicated concentrations of fluvastatin and/or bortezomib for 48 hours. Thirty minutes prior to imaging, cells were stained with Hoechst-33342 (8 nM; Cell Signalling), tetramethylrhodamine ethyl ester perchlorate (TMRE) (10 μM and 4 μM for H929 and EJM, respectively; Life Technologies), and Annexin V conjugated to Alexa Fluor 488 (1:2,500 and 1:5,000 for H929 and EJM, respectively; prepared in-house as described previously[1]). Cells were imaged using an Opera Phenix automated confocal microscope (PerkinElmer) with a 20x air objective. Nine different fields of view for each well were acquired. Three technical replicates per treatment condition were performed for each of three independent experiments. Image acquisition, calculation of intensity features for each channel and image analysis were performed using the Harmony high-content imaging and analysis software v4.9 (PerkinElmer). Hoechst-33342 staining was used to identify all cells in the well and to measure morphology features related to cell death (e.g. nuclear condensation), TMRE was used to identify cells with either active (polarized) or depolarized mitochondria, and Annexin V was used to identify cells with externalized phosphatidylserine (apoptotic cells). The image data sets were analyzed using a linear classifier to determine the percentage of dead MM cells per treatment condition. Briefly, training sets consisting of approximately 20 cells

per population ("viable" or "dead") were generated, and phenotypic parameters and intensity features were calculated for every cell. The software then calculated a multi-parametric linear classifier optimized to discriminate between "viable" and "dead" cells using PhenoLOGIC^{TM}, and the percentage of "dead" cells was calculated for each treatment condition.

RNA interference

Three independent short hairpin RNAs (shRNAs) against *FGFR3* were designed using The RNAi Consortium (TRC) Genetic Perturbation Platform (portals.broadinstitute.org/gpp/public) and cloned into the doxycycline-inducible pLKO shRNA lentiviral system (see below for shFGFR3 sequences). The 5' and 3' shRNA constructs for MMSET and the corresponding control shRNA were characterized previously[2]. HEK-293Tv cells were co-transfected with the shRNA constructs, pMD2.G and psPAX2 via calcium phosphate transfection. Viral supernatants were harvested 48 hours post-transfection. KMS11 cells were transduced with the lentiviral supernatants in the presence of 8 μg/mL polybrene, after which they were selected in 1 μg/mL puromycin.

**shFGFR3**	**Sequence (5' to 3')**
#1	TRCN0000000372:<br>*Forward*:<br>CCGGTGCGTCGTGGAGAACAAGTTTCTCGAGAAACTTGTTCTCCACGACGCATTTTTG<br>*Reverse*:<br>AATTCAAAAATGCGTCGTGGAGAACAAGTTTCTCGAGAAACTTGTTCTCCACGACGCA
#2	TRCN0000196809:<br>*Forward*:<br>CCGGGTGATTCCAGTGAAGATATTTCTCGAGAAATATCTTCACTGGAATCACTTTTTG<br>*Reverse*:<br>AATTCAAAAAGTGATTCCAGTGAAGATATTTCTCGAGAAATATCTTCACTGGAATCAC
#3	TRCN0000195570:<br>*Forward*:<br>CCGGCGTGATGAAGATCGCAGACTTCTCGAGAAGTCTGCGATCTTCATCACGTTTTTG<br>*Reverse*:<br>AATTCAAAAACGTGATGAAGATCGCAGACTTCTCGAGAAGTCTGCGATCTTCATCACG

Differential analysis of basal mRNA expression

RNA sequencing (RNA-seq) data for 14 MM cell lines (see Figure 1B) were previously generated by Dr. Jonathan Keats (Translational Genomics Research Institute, Phoenix, AZ, USA) and made available through the Multiple Myeloma Research Foundation (MMRF). The sequencing depth ranged from 48-71 (median 61) million reads per cell line, with 83 base pair read length. STAR (v2.5.2b)[3] was used to align the transcript reads to the reference genome, using the GRCh38 reference[4] and GENCODE v26 annotation[5]. RSEM[6] was used to estimate gene level counts for the expression of each gene annotated in GENCODE v26. Cell lines were stratified into "statin-sensitive" or "statin-insensitive" MM cell lines based on previously published data[7]. Differential expression analysis between the statin-sensitive and insensitive groups was conducted using the DESeq2 package (v1.24.0)[8]. Genes were filtered to those annotated as protein-coding to reduce multiple testing burden. Prior to differential expression, a Cook's Distance threshold (samples with Cook's distance > 4/11 removed) was used to filter out genes where differential expression was driven primarily by outlier expression in a small proportion of cell lines. Comparison was done between the mean counts of the sensitive and insensitive group, with sample-specific size factors and gene-specific dispersion estimated using the default DESeq2 methods, and a Negative Binomial Wald test was applied to calculate the significance of each observed fold change. A Bonferroni correction for multiple testing was applied and alpha = 0.05 was used to call differentially expressed genes.

Differential analysis of mRNA expression in MM cell lines before and after statin treatment

H929 and KMS11 cells were seeded at 750,000 cells/well into 6-well plates and treated with either ethanol (solvent control) or 2 μM fluvastatin for 24 hours, a timepoint prior to cell death at this concentration of fluvastatin. Total RNA was isolated from two biological replicates using the RNeasy Plus Kit (Qiagen) as per the manufacturer's instructions. RNA libraries were prepared using the TruSeq Stranded Total RNA kit (Illumina), with RiboZero Gold (Illumina) for depletion of ribosomal RNA. RNA libraries were paired-end sequenced (75 base pair read length) using an Illumina NextSeq500 to a target read depth of 40 million reads (actual observed read depths of 28-58 million, median 43 million). Processing of RNA-seq reads and differential expression analysis between the ethanol- and fluvastatin-treated groups were performed as described above. For each gene, comparison was done between the mean counts before and after treatment, for the two cell

lines together. This was done to assess perturbations common to the sensitive phenotype, rather than cell line-specific perturbations. After FDR multiple testing correction, a q-value threshold of 0.05 was used to call significant differentially expressed genes between the two groups.

Gene ontology (GO) analysis

Lists of genes upregulated or downregulated after fluvastatin treatment were subjected to GO analysis using the Enrichr tool (https://amp.pharm.mssm.edu/Enrichr/)[9]. The top 10 "GO Biological Process 2018" GO terms, ranked by combination score, were plotted. The combined enrichment score is comprised of both the p-value (Fischer's exact test) and z-score [combined score = ln(p)*z)].

Gene set enrichment analysis (GSEA)

All available gene expression data between ethanol control- and 2 μM fluvastatin-treated H929 and KMS11 cells were first ranked by $\log_2$(fold change) and then used for GSEA[10] with a list of 472 previously reported ATF4 target genes[11]. GSEA software version 4.0.2 software was used.

Statistical analysis

All graphs depict the mean of at least three biological replicates, with error bars representing the standard deviation (SD) of the mean, unless otherwise indicated in the figure legend. Statistical analyses were performed using GraphPad Prism version 7 or 8 software. For each figure, the sample size and statistical test performed are noted in the legend. Sample sizes were chosen based on previous publications[7,12,13]. For the one-way ANOVA analyses, a Brown-Forsythe test was performed to test for equal variance. If the data did not pass the equal variance test (alpha = 0.05), then a Kruskal-Wallis test was performed instead of a one-way ANOVA (e.g. Figure 5A). A p-value of < 0.05 was considered statistically significant. Adjustments for multiple comparisons were performed where appropriate. Statistical analysis of differential gene expression was done using standard methods, as described above.

SUPPLEMENTAL REFERENCES

1 Logue SE, Elgendy M, Martin SJ. Expression, purification and use of recombinant annexin V for the detection of apoptotic cells. *Nat Protoc* 2009; **4**: 1383–1395.

2 Martinez-Garcia E, Popovic R, Min D-J, Sweet SMM, Thomas PM, Zamdborg L *et al.* The MMSET histone methyl transferase switches global histone methylation and alters gene expression in t(4;14) multiple myeloma cells. *Blood* 2011; **117**: 211–20.

3 Dobin A, Davis CA, Schlesinger F, Drenkow J, Zaleski C, Jha S *et al.* STAR: Ultrafast universal RNA-seq aligner. *Bioinformatics* 2013; **29**: 15–21.

4 Schneider VA, Graves-Lindsay T, Howe K, Bouk N, Chen HC, Kitts PA *et al.* Evaluation of GRCh38 and de novo haploid genome assemblies demonstrates the enduring quality of the reference assembly. *Genome Res* 2017; **27**: 849–864.

5 Frankish A, Diekhans M, Ferreira AM, Johnson R, Jungreis I, Loveland J *et al.* GENCODE reference annotation for the human and mouse genomes. *Nucleic Acids Res* 2019; **47**: D766–D773.

6 Li B, Dewey CN. RSEM: accurate transcript quantification from RNA-Seq data with or without a reference genome. *BMC Bioinformatics* 2011; **12**: 323.

7 Wong WW-L, Clendening JW, Martirosyan A, Boutros PC, Bros C, Khosravi F *et al.* Determinants of sensitivity to lovastatin-induced apoptosis in multiple myeloma. *Mol Cancer Ther* 2007; **6**: 1886–1897.

8 Love MI, Huber W, Anders S. Moderated estimation of fold change and dispersion for RNA-seq data with DESeq2. *Genome Biol* 2014; **15**: 550.

9 Kuleshov M V., Jones MR, Rouillard AD, Fernandez NF, Duan Q, Wang Z *et al.* Enrichr: a comprehensive gene set enrichment analysis web server 2016 update. *Nucleic Acids Res* 2016; **44**: W90–W97.

10 Subramanian A, Tamayo P, Mootha VK, Mukherjee S, Ebert BL, Gillette MA *et al.* Gene set enrichment analysis: A knowledge-based approach for interpreting genome-wide expression profiles. *Proc Natl Acad Sci U S A* 2005; **102**: 15545–15550.

11 Han J, Back SH, Hur J, Lin YH, Gildersleeve R, Shan J *et al.* ER-stress-induced transcriptional regulation increases protein synthesis leading to cell death. *Nat Cell Biol* 2013; **15**: 481–490.

12 Punnoose EA, Leverson JD, Peale F, Boghaert ER, Belmont LD, Tan N *et al.* Expression profile of BCL-2, BCL-XL, and MCL-1 predicts pharmacological response to the BCL-2 selective antagonist venetoclax in multiple myeloma models. *Mol Cancer Ther* 2016; **15**: 1132–1144.

13 Lee JS, Roberts A, Juarez D, Vo TTT, Bhatt S, Herzog LO *et al.* Statins enhance efficacy of venetoclax in blood cancers. *Sci Transl Med* 2018; **10**: eaaq1240.

SUPPLEMENTAL FIGURES & TABLES

Table S1: List of differentially expressed genes between statin-sensitive and insensitive MM cell lines (related to Figure 1C).

ENSG ID	log_2(fold change)	p-value	Adjusted p-value	Gene symbol
ENSG00000125910.5	-8.6036	0.00000001	0.00010640	S1PR4
ENSG00000170542.5	-6.1708	0.00000151	0.01512221	SERPINB9
ENSG00000115085.13	-4.9004	0.00000015	0.00146117	ZAP70
ENSG00000140403.12	-4.4468	0.00000041	0.00407861	DNAJA4
ENSG00000008513.14	-2.3514	0.00000414	0.04140494	ST3GAL1
ENSG00000156509.13	-1.9108	0.00000466	0.04666383	FBXO43
ENSG00000205213.13	-1.8581	0.00000466	0.04663486	LGR4
ENSG00000179715.12	4.6634	0.00000096	0.00956768	PCED1B
ENSG00000109113.18	5.5186	0.00000323	0.03235753	RAB34
ENSG00000259803.6	5.7334	0.00000093	0.00933686	SLC22A31
ENSG00000068078.17	6.6313	0.00000149	0.01488581	FGFR3
ENSG00000174307.6	7.0017	0.00000320	0.03198370	PHLDA3
ENSG00000204983.13	7.8526	0.00000248	0.02482769	PRSS1
ENSG00000102287.18	8.2664	0.00000002	0.00021728	GABRE
ENSG00000112837.16	8.5996	0.00000067	0.00669836	TBX18
ENSG00000151962.7	9.8314	0.00000246	0.02467121	RBM46

A

Patient	t(4;14) status	Viable CD138+ cells (%)		
		EtOH	2.5 µM Fluva	5 µM Fluva
1	+	18.1	N/A	5.2
2	+	15.8	10.1	6.3
3	-	10.3	6.0	4.8
4	-	15.6	11.0	10.0
5	+	15.7	13.8	11.6
6	-	46.3	44.7	37.5
7	-	31.0	28.1	27.6
8	-	33.3	25.5	29.8

B

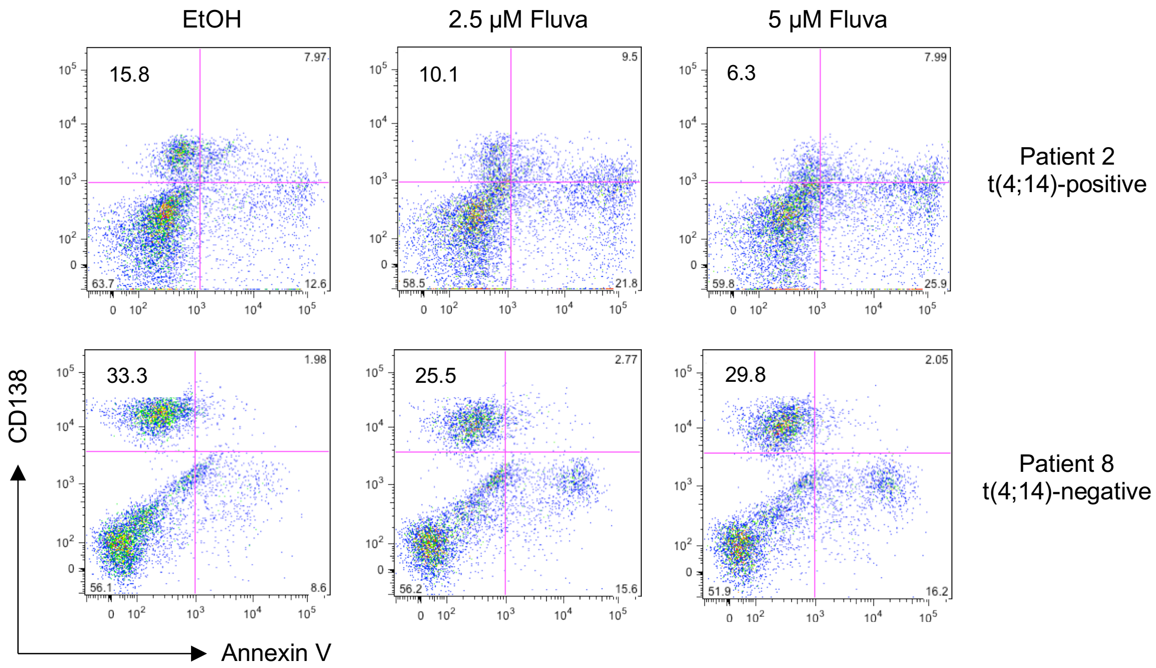

Figure S1: Primary t(4;14)-positive MM cells undergo apoptosis in response to fluvastatin *ex vivo*. (**A**) Primary MM cells were cultured in the presence of ethanol (EtOH; solvent control), 2.5 µM fluvastatin or 5 µM fluvastatin. After 72 hr, cells were labelled with PE-conjugated anti-CD138 and FITC-conjugated Annexin V and then analyzed by flow cytometry. The percentages of viable MM cells (CD138+/Annexin V-) for each patient are summarized, together with the t(4;14) translocation status of each patient (confirmed by fluorescence *in situ* hybridization). (**B**) Representative plots for one t(4;14)-positive and one t(4;14)-negative patient.

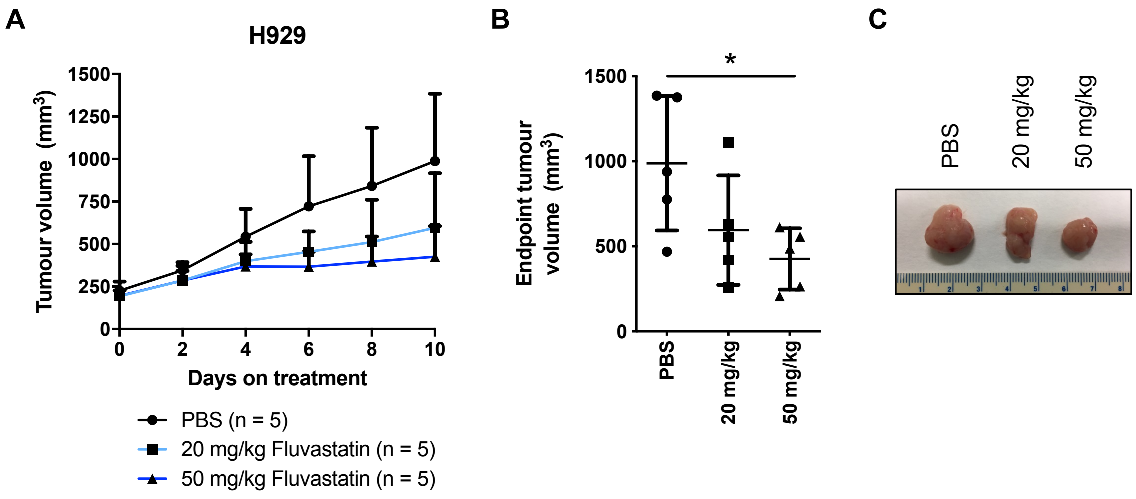

Figure S2: Fluvastatin delays tumour growth *in vivo* in a t(4;14)-positive xenograft model. (**A**) NOD/SCID mice bearing t(4;14)-positive H929 xenografts were randomized to receive PBS (vehicle control), 20 mg/kg/day fluvastatin or 50 mg/kg/day fluvastatin by oral gavage once tumours reached ~200 mm^3 in volume. Tumour measurement assessments were not blinded. The data are represented as the mean + SD, n = 5 mice per treatment group. (**B**) Tumour volumes after 10 days of treatment. The data are represented as the mean ± SD, n = 5 mice per treatment group, *$p < 0.05$ (one-way ANOVA with Bonferroni's multiple comparisons test, where each group was compared to the PBS control group). (**C**) Representative tumour images at endpoint (day 10).

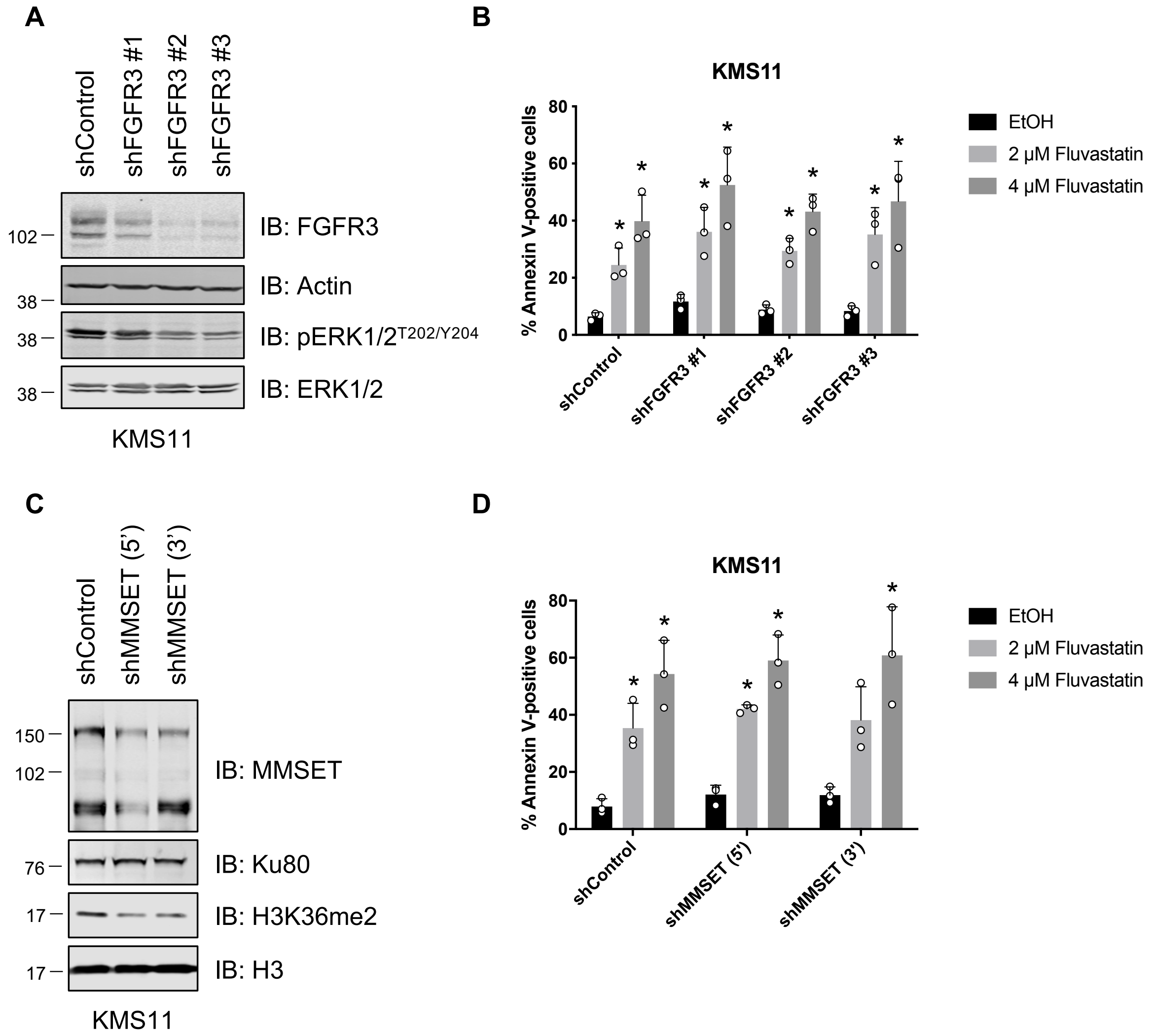

Figure S3: Fluvastatin sensitivity in t(4;14)-positive KMS11 cells is independent of FGFR3 and MMSET. (**A**) KMS11 cells expressing inducible shRNAs against *FGFR3* were induced for 48 hr with 1 µg/mL doxycycline, and then protein was isolated to assay for FGFR3 and ERK1/2 expression by immunoblotting. (**B**) shControl or shFGFR3 cells were treated with ethanol (EtOH) as a solvent control, 2 µM fluvastatin or 4 µM fluvastatin for 48 hr in the presence of 1 µg/mL doxycycline and apoptosis was determined by Annexin V staining. The data are represented as the mean + SD, n = 3, $*p < 0.05$ (one-way ANOVA with Bonferroni's multiple comparisons test, where each treatment group was compared to their respective EtOH control group). (**C**) KMS11 cells expressing inducible shRNAs against *MMSET* were induced for 7 days with 2 µg/mL doxycycline, and then protein was isolated to assay for MMSET and H3K36me2 expression by immunoblotting. (**D**) shControl or shMMSET cells were pre-treated with 2 µg/mL doxycycline for 5 days, and then treated with EtOH, 2 µM fluvastatin or 4 µM fluvastatin for an additional 48 hr in the presence of 2 µg/mL doxycycline. Apoptosis was determined by Annexin V staining. The data are represented as the mean + SD, n = 3, $*p < 0.05$ (one-way ANOVA with Bonferroni's multiple comparisons test, where each treatment group was compared to their respective EtOH control group).

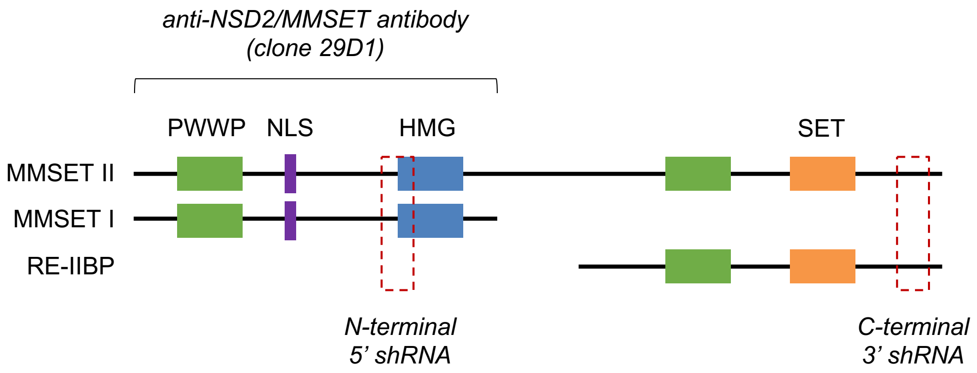

Figure S4: Schematic of MMSET proteins and reagents. Complex alternative splicing and alternative promoter usage gives rise to three major protein products from the *MMSET* gene (MMSET II, MMSET I and RE-IIBP). Both MMSET II and MMSET I possess a PWWP domain, nuclear localization signal (NLS) and high mobility group (HMG) box domain; however, MMSET I lacks the SET domain that is required for MMSET's histone methyltransferase activity. The 5' *MMSET* shRNA used in this study depletes both MMSET II and MMSET I, whereas the 3' shRNA depletes MMSET II and RE-IIBP (the product of an independent transcript that initiates from exon 15 of the *MMSET* gene). The mouse monoclonal antibody (clone 29D1) used in this study to detect MMSET by immunoblotting recognizes the N-terminal portion of the protein, and therefore detects MMSET II and MMSET I, but not RE-IIBP. The MMSET schematic was adapted from Martinez-Garcia *et al.*[2], where the shRNA constructs and antibody are further described.

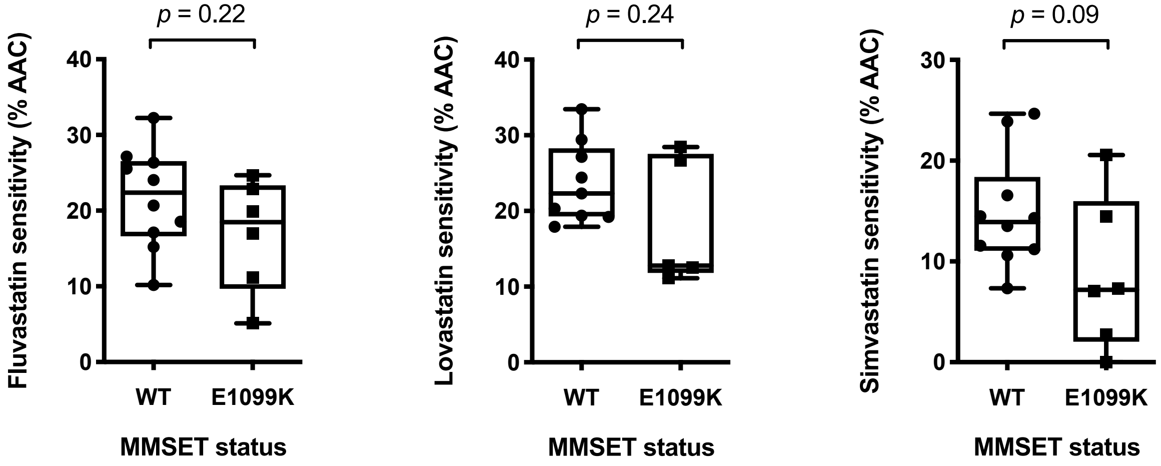

Figure S5: Statin sensitivity is not associated with the MMSET E1099K mutation in ALL cell lines. Sensitivity to fluvastatin, lovastatin and simvastatin in MMSET wildtype or mutant (E1099K) ALL cell lines mined from the CTRPv2 database. Percent area above the drug dose-response curve (% AAC) values are plotted as a box plot with whiskers representing minimum and maximum values. p-values were derived from Wilcoxon rank sum tests (unpaired, two-tailed), comparing MMSET wildtype and mutant (E1099K) cell lines.

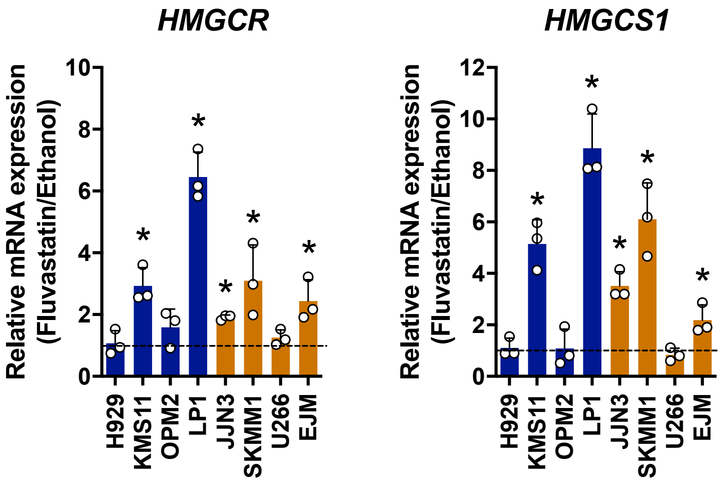

Figure S6: t(4;14) translocation status is not associated with impaired feedback regulation of the MVA pathway. MM cell lines were treated with 4 μM fluvastatin or ethanol as a solvent control for 16 hr, and then RNA was isolated to assay for *HMGCR* and *HMGCS1* expression by qRT-PCR. Expression data are normalized to *RPL13A* and plotted relative to the ethanol control. The data are represented as the mean + SD, n = 3, *p < 0.05 (Student *t* test, unpaired, two-tailed). Blue and orange bars represent t(4;14)-positive and t(4;14)-negative cell lines, respectively.

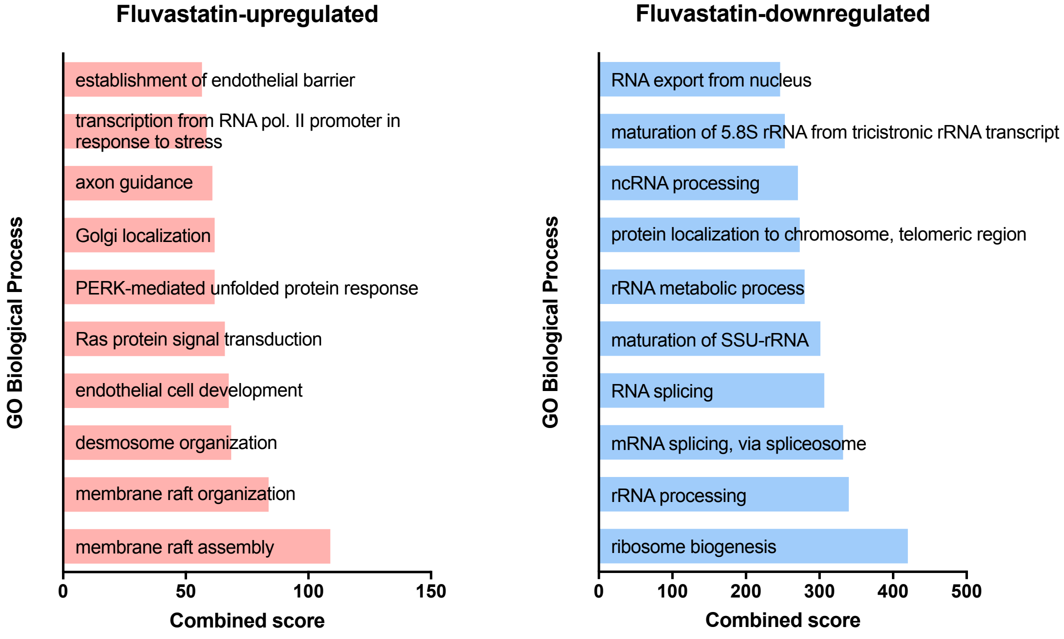

Figure S7: Gene ontology terms associated with response to fluvastatin treatment in t(4;14)-positive MM cell lines. Gene ontology (GO) analysis of top 10 biological processes upregulated or downregulated following 2 μM fluvastatin treatment for 24 hr in H929 and KMS11 cells. The combined enrichment score is comprised of both the p-value (Fischer's exact test) and z-score [combined score = ln(p)*z)].

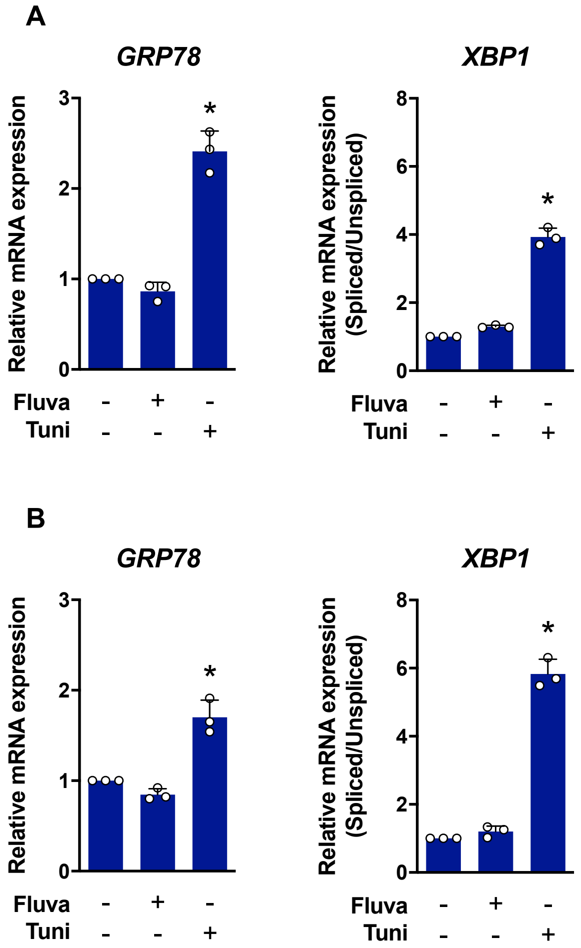

Figure S8: Fluvastatin does not induce other unfolded protein response (UPR) markers in t(4;14)-positive cells. (**A**) KMS11 or (**B**) LP1 cells were treated with solvent controls, 4 μM fluvastatin or 0.5 μg/mL tunicamycin for 24 hr, after which RNA was isolated for qRT-PCR. Expression of the UPR-associated gene *GRP78* and *XBP1* splicing were evaluated, and RNA expression was normalized to *RPL13A*. For *XBP1* splicing, primers that recognize either spliced or unspliced *XBP1* were used, and data are expressed as spliced/unspliced *XBP1*. The data are represented as the mean + SD, n = 3, *p < 0.05 (one-way ANOVA with Bonferroni's multiple comparisons test, where each group was compared to the solvent controls group).

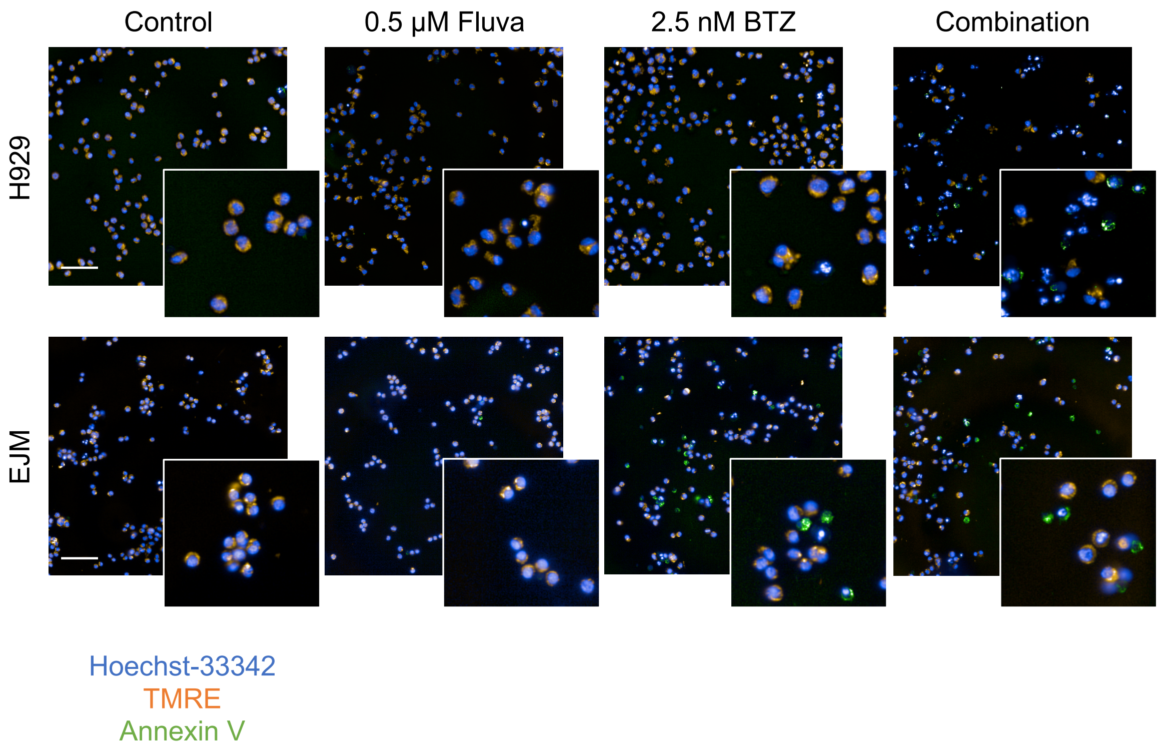

Figure S9: Representative images of MM cells treated with fluvastatin and/or bortezomib. H929 cells (top row) and EJM cells (bottom row) were treated with solvent controls, 0.5 µM fluvastatin, 2.5 nM bortezomib (BTZ) or the combination for 48 hours. Cells were then stained with Hoechst-33342 (blue), TMRE (orange) and Annexin V-Alexa Fluor 488 (green). Stained cells were imaged by confocal microscopy. Scale bars = 100 µm.

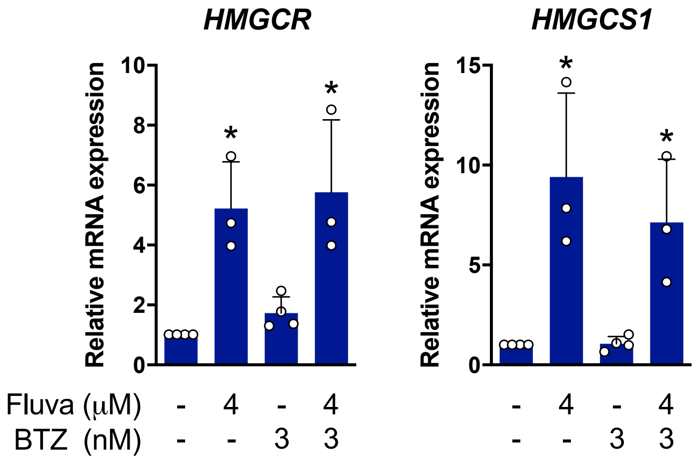

Figure S10: Bortezomib does not inhibit the fluvastatin-induced, sterol-regulated feedback mechanism of the MVA pathway. LP1 cells were treated with solvent controls, 4 μM fluvastatin, 3 nM bortezomib (BTZ) or the combination for 24 hr, after which RNA was isolated. Expression of the SREBP2 target genes *HMGCR* and *HMGCS1* was evaluated by qRT-PCR, and their expression normalized to *RPL13A*. The data are represented as the mean + SD, n = 3, *p < 0.05 (one-way ANOVA with Bonferroni's multiple comparisons test, where each group was compared to the solvent controls group).
